# Towards an Improved Standard in Penile Duplex Doppler Ultrasonography: A Randomized Crossover Trial of 3D Virtual Glasses for Audiovisual Sexual Stimulation

**DOI:** 10.3390/jcm14217762

**Published:** 2025-11-01

**Authors:** Tae Young Park, Jae Young Hwang, Seong Woo Yun, Hyun Jung Jin, Sung Goo Yoon, Tae Il Noh, Ji Sung Shim, Sung Gu Kang, Seok Ho Kang, Min Gu Park

**Affiliations:** Department of Urology, Korea University Anam Hospital, Korea University College of Medicine, Seoul 02841, Republic of Korea; tyoung93@naver.com (T.Y.P.); peterhjy@naver.com (J.Y.H.); statea11@naver.com (S.W.Y.); hjjin51893@naver.com (H.J.J.); seong-goo@naver.com (S.G.Y.); mdksh@korea.ac.kr (S.H.K.)

**Keywords:** 3D virtual glasses, audiovisual sexual stimulation, penile duplex Doppler ultrasound

## Abstract

**Background/Objectives**: We compared the efficacy of audiovisual sexual stimulation (AVSS) using 3D virtual glasses with a desktop monitor. **Methods**: In this randomized crossover study, 60 patients with ED underwent two penile duplex Doppler ultrasound sessions 1 week apart, each after intracavernosal prostaglandin E1 injection. Patients were randomly assigned to receive AVSS via 3D virtual glasses or a desktop monitor in the first session, with crossover in the second. We measured the resistive index (RI), erectile hardness score (EHS), peak systolic velocity (PSV), anxiety (State-Trait Anxiety Inventory), and satisfaction (visual analog scale). **Results**: 3D virtual glasses were associated with superior outcomes. The linear mixed models analysis showed higher adjusted mean RI (*p* < 0.001), PSV (*p* < 0.001), and satisfaction (*p* < 0.001) for 3D glasses. Generalized estimating equations analysis showed patients were 6.68 times more likely to achieve functional erection (EHS ≥ 3) with 3D glasses (odds ratio 6.68, 95% confidence interval [2.54, 17.55], *p* < 0.001). Anticipatory anxiety before subsequent examinations was lower with virtual glasses (*p* < 0.001). **Conclusions**: AVSS with 3D virtual glasses is associated with superior hemodynamic parameters and clinical responses consistent with reduced false-positive diagnoses. It also lowers anticipatory anxiety for subsequent procedures, enhancing patient experience.

## 1. Introduction

Erectile dysfunction (ED) is defined as the recurrent failure to attain and maintain an erection adequate for satisfactory sexual activity [1]. It is a prevalent and clinically significant disorder that severely affects the quality of life worldwide [2]. The condition has multiple underlying mechanisms, and a comprehensive diagnostic evaluation is imperative for selecting the optimal treatment strategy and improving therapeutic outcomes [3,4,5]. While many etiologies exist, ED is predominantly vasculogenic, accounting for approximately 60–80% of cases [6,7]. The gold standard for evaluating penile blood flow in patients with ED is the penile duplex Doppler ultrasound (PDDU) following an intracavernous injection (ICI) of a vasoactive agent [8]. For accurate evaluation, adequate cavernosal smooth muscle relaxation is essential [9,10]. Nonetheless, increased sympathetic activity due to patient anxiety can compromise the relaxation process, potentially leading to false-positive results [11].

Therefore, various methods have been introduced to enhance relaxation, with audiovisual sexual stimulation (AVSS) demonstrating efficacy and feasibility in several studies [12,13,14,15]. Montorsi et al. compared the effect of a second vasoactive injection versus an initial injection combined with genital and audiovisual sexual stimulation [13]. They reported that the group receiving AVSS achieved significantly higher maximum rigidity compared to the redosing group (87% vs. 47%, respectively). Katlowitz et al. investigated the potentiation of drug-induced erections with audiovisual sexual stimulation [14]. Their study found that the majority of patients demonstrated improved penile hemodynamic parameters after the addition of AVSS. Nevertheless, the traditional AVSS has practical limitations due to the presence of clinicians during the examination, which may cause patient discomfort, compromise privacy, and consequently inhibit erectile responses [16,17]. This tendency may contribute to a lack of reproducibility in PDDU measurements, where results vary across repeated examinations, as investigated in studies such as that by Mikkonen et al. [18]. Given these limitations of conventional methods, a growing trend exists in men’s health to develop and apply novel technologies for more objective and quantitative assessments of erectile function [19,20]. This move toward technological innovation underscores the clinical need for methods that can provide more accurate data while improving the patient experience. Among these innovations, virtual glasses have shown efficacy in reducing patient anxiety during medical procedures and treatments [21]. Therefore, novel AVSS utilizing virtual glasses has been introduced, and preliminary studies have suggested its effectiveness [16,22,23]. Using a controllable AVSS system with a glasses-type video monitor and earphones (Eye-Trek^®^ head-mounted display, Olympus, Japan), Kuo et al. found that combining it with an injection significantly improved peak systolic velocity (PSV) [16]. Park et al. conducted an early study demonstrating that using virtual glasses for AVSS during penile ultrasonography was effective [22]. Pescatori et al. showed that virtual glasses were highly effective for AVSS, as 80% of patients achieved complete smooth muscle relaxation, compared to only 33% without them [23].

Despite these promising preliminary findings, two critical gaps remain. First, no study has directly compared virtual glasses with conventional AVSS to prove superiority in diagnostic accuracy or patient satisfaction. Second, the devices used in prior studies were older, less advanced models.

We therefore hypothesized that a technologically advanced, immersive 3D modality would yield superior results. We predicted it would more effectively induce cavernosal relaxation and reduce false-positive diagnoses. Furthermore, we postulated that this modality would improve patient satisfaction and lower anxiety levels.

This study aimed to directly compare the efficacy of AVSS using 3D virtual glasses with that of the traditional AVSS method, considering key outcomes such as patient anxiety, satisfaction, smooth muscle relaxation, and the incidence of false-positive diagnosis.

## 2. Materials and Methods

This study was designed as a prospective, randomized, single-center, crossover trial.

Sixty consecutive patients who presented with a chief complaint of ED were recruited. Eligible patients were males aged ≥ 20 years who reported an inability to achieve or maintain an erection sufficient for satisfactory sexual performance for at least 6 months and had a baseline International Index of Erectile Function-5 (IIEF-5) score of 21 or lower. Written informed consent was obtained from all participants before enrollment. The exclusion criteria were daily use of a phosphodiesterase type 5 (PDE5) inhibitor, on-demand use of a PDE5 inhibitor on the examination day, priapism history, and current anticoagulant therapy use. Patients with comorbidities known to affect erectile function (e.g., diabetes mellitus, major psychiatric disorders) were not excluded from the study.

The required sample size was determined by an a priori power analysis informed by the results of a previous randomized crossover trial by Carneiro et al. [15], which served as the best available reference for estimating the effect of AVSS. We used the Resistive Index (RI) as the primary outcome. The Carneiro et al. study data indicated a mean RI of approximately 1.02 (pooled from reported means of 1.04 ± 0.14 and 1.00 ± 0.17) without AVSS and approximately 1.09 (pooled from 1.04 ± 0.20 and 1.13 ± 0.25) with AVSS. We note that RI values > 1.0 are possible in this context, as the RI calculation (peak systolic velocity [PSV]-end-diastolic velocity [EDV])/PSV can exceed 1.0 when EDV is negative (reversed flow). Ideally, a power calculation for a crossover design should use the within-subject standard deviation (SD of the difference), but this value was not reported in the reference study. We therefore had to conservatively estimate the effect size using the reported pooled standard deviation (approximately 0.19) and the difference between the means (0.07), yielding an approximate effect size (Cohen’s d) of 0.37. To detect this estimated effect size (d ≈ 0.37) with a statistical power of 80% at a two-sided significance level of 0.05 using a paired t-test, a minimum sample size of 60 patients was calculated. Our enrollment of 60 patients precisely met this calculated target.

The patients were randomized in a 1:1 ratio into two sequence groups (Group I: Desktop first; Group II: 3D Glasses first). The randomization sequence was generated using a computer-based random number generator. To ensure allocation concealment, the sequence was managed by a central research coordinator who was not involved in patient enrollment or outcome assessment. After a participant was confirmed eligible and provided written informed consent, the enrolling physician contacted this coordinator to receive the allocation assignment.

All patients underwent two PDDU sessions, conducted 1 week apart. This 1-week interval was chosen as a standard “washout” period, considered more than sufficient to prevent any physiological carryover from the prostaglandin E1 injection and to minimize recall or adaptation bias. The sessions were held in a quiet, temperature-controlled private room with dimmed lighting to ensure patient comfort and minimize external distractions. Each session was preceded by an intracavernosal injection of prostaglandin E1 (10–20 μg) within 5 min before PDDU. For the virtual glasses modality, the participants wore a glasses-type video monitor (Samsung Gear VR SM-R324; Samsung, Seoul, Republic of Korea) equipped with integrated earphones to provide an immersive experience and minimize external distractions. For the traditional modality, a standard 24-inch desktop monitor was used. To ensure stimulus consistency, the videos consisted of commercially available, standardized pornographic material featuring heterosexual content. To prevent content-based bias, one video file was designated for the first session, and a different file was selected for the second. All participants were exposed to the same video file in each session, ensuring the content remained identical across both the 3D virtual glasses and desktop monitor conditions. During the duplex scan, patients were placed in a supine position, and an ultrasound probe (ACCUVIX A30; Samsung Medison, Seoul, Republic of Korea) was placed ventrally at the penoscrotal junction. The cavernous arterial blood flow was measured along the longitudinal plane for 30 min, and PSV and end-diastolic velocity (EDV) were recorded for analysis. The RI, defined as (PSV − EDV)/PSV, was also calculated for analysis.

The criteria for normal vascular function were based on established hemodynamic standards [15]: cavernous arterial inflow was considered adequate with a mean PSV > 30 cm/s, and veno-occlusive function was considered normal with an RI > 0.9 and an EDV < 5 cm/s. These criteria were applied to the peak measurements obtained during the 30 min examination period.

Baseline erectile function was assessed using the IIEF. Clinical responses (erectile hardness score [EHS]) were measured with visual inspection and manual examination (Grade 1: tumescence only; Grade 2: rigid but not sufficient for penetration; Grade 3: rigid for penetration but not fully rigid; Grade 4: fully rigid).

Anxiety levels were assessed using the State-Trait Anxiety Inventory-State (STAI-S) subscale. The questionnaire was administered at two time points: (T1) immediately after the first PDDU session and (T2) just before the second session, 1 week later. Patient satisfaction and willingness to undergo a repeat procedure were self-assessed after each PDDU using a visual analog scale (VAS, 0–10).

Primary and secondary continuous outcomes (PSV, EDV, RI, mean EHS, and VAS scores) were analyzed using linear mixed models (LMM) to account for the randomized crossover design. The binary outcome of functional erection (EHS ≥ 3) was analyzed using generalized estimating equations (GEE) with a binomial distribution and logit link. These models included modality (Desktop vs. Virtual Glasses), period (Session 1 vs. 2), and sequence (Group I vs. II) as fixed effects, allowing assessment of the main modality effect and any potential carryover effect.

STAI anxiety scores, collected at two time points (after session 1 and before session 2), were analyzed using independent group comparisons for each time point. Normality was assessed using the Shapiro–Wilk test, and homogeneity of variance was evaluated using Levene’s test. The STAI data met the assumption of normality but violated the assumption of equal variances (Levene’s test, *p* < 0.05). Consequently, Welch’s *t*-test was employed for these comparisons.

All statistical analyses were conducted using SPSS software (version 23.0; IBM Corp., Armonk, NY, USA). A *p*-value < 0.05 was considered to indicate statistical significance. This study was approved by the Institutional Review Board (IRB No. 2019-07-007).

## 3. Results

A total of 60 patients were enrolled and completed the crossover trial. No significant difference was observed in age or baseline IIEF score between the two groups. The comprehensive results of the analysis are presented in Table 1.

The analysis demonstrated a significant modality effect favoring 3D virtual glasses across all primary and secondary outcomes. For our a priori primary endpoint, the RI, the LMM analysis revealed a highly significant modality effect (*p* < 0.001). The adjusted mean RI was 0.90 with 3D virtual glasses compared to 0.87 with the desktop monitor (Net Effect: +0.03).

Secondary hemodynamic outcomes also strongly favored the 3D virtual glasses. The adjusted mean PSV was significantly higher with 3D virtual glasses (35.27 cm/s) than with the desktop (29.30 cm/s), showing a large net effect of +5.97 cm/s (*p* < 0.001). Conversely, the adjusted mean EDV was lower with 3D virtual glasses (3.36 cm/s) compared to the desktop (3.65 cm/s) (Net Effect: −0.29 cm/s, *p* = 0.013). For both RI and PSV, a significant period effect was detected (*p* = 0.011 and *p* = 0.008, respectively), confirming the necessity of the mixed-effects model. No significant carryover effect was found for any hemodynamic variable (*p* > 0.05).

Patient-reported outcomes showed a clear preference for the 3D virtual glasses. The adjusted mean satisfaction (VAS) score was 5.60 for 3D virtual glasses, significantly higher than the 3.47 for the desktop (Net Effect: +2.13, *p* < 0.001). Willingness to undergo a repeat procedure (VAS) was also significantly higher for the 3D virtual glasses (*p* < 0.001).

The analysis of anxiety (STAI-S) scores revealed a significant carryover effect (*p* < 0.001) in the LMM (Table 1), invalidating the main modality effect estimate. Therefore, a separate parallel-group analysis was conducted, as shown in Table 2.

A Welch’s *t*-test of STAI-S scores measured after the first session showed no statistically significant difference between the group that used the desktop (25.47 ± 6.82) and the group that used 3D virtual glasses (23.13 ± 3.93) (*p* = 0.263).

Nevertheless, a highly significant difference was observed in the anticipatory anxiety measured before the second session. Patients who had first experienced the desktop modality (Group 1) reported significantly higher anxiety (mean STAI-S = 38.13 ± 5.60) about the upcoming procedure compared to those who had first experienced the 3D virtual glasses modality (Group 2) (Mean STAI-S = 25.87 ± 3.07) (*p* < 0.001).

## 4. Discussion

The use of PDDU following an ICI of a vasoactive agent has been the gold standard for evaluating vasculogenic ED since its introduction [8]. However, subsequent research has shown that accurate test interpretation is critically dependent on adequate relaxation of the cavernosal smooth muscle [7]. It is well established that patient anxiety can impair this relaxation through increased sympathetic activity, potentially resulting in misdiagnosis during PDDU testing for ED, particularly in young adults [11,24]. Although methods such as vasodilator redose have been utilized to overcome this limitation, they have proven to be largely ineffective [10,25]. Contrastingly, the AVSS has emerged as a promising alternative to achieve this goal [26]. According to early studies by Montorsi et al. and Katlowitz et al., combining AVSS with an initial intracavernous injection enhances erectile response, resulting in significantly higher maximum rigidity compared to repeat injections and improved overall hemodynamic parameters [13,14]. Building on these findings, a recent randomized crossover trial by Carneiro et al. [15] further underscored the clinical impact of AVSS. Their results showed that it significantly improved veno-occlusive parameters such as EDV and RI. This improvement also led to a diagnostic reclassification from vasculogenic ED to normal in 10% of patients. Nevertheless, conventional AVSS modalities using a desktop monitor present some challenges. The presence of a clinician for ultrasound examination can cause patient discomfort and anxiety, potentially inhibiting the erectile response [16,17], highlighting the need for a more immersive method to reduce patient anxiety by blocking external stimuli and minimizing awareness of the presence of the examiner. Consequently, AVSS with virtual glasses has been proposed as a potential solution [23]. Nonetheless, previous studies have not directly compared virtual glasses with traditional modalities, such as desktop monitors [16,22,23]. To our knowledge, this is the first randomized controlled trial to conduct a direct comparison to determine the optimal AVSS method.

Here, the AVSS with 3D virtual glasses yielded significantly superior erectile responses compared to the desktop monitor. Similarly, patient-reported outcomes, including satisfaction and willingness to repeat the procedure, were significantly increased when patients used 3D virtual glasses.

The randomized crossover design was chosen to minimize interpatient variability. A key consideration in this design is the potential for confounding factors such as period (adaptation) and carryover effects. To address this, our statistical analysis employed LMM to model these factors (Table 1). This analysis yielded a crucial finding: first, a significant period effect was identified for key hemodynamic outcomes such as RI (*p* = 0.011) and PSV (*p* = 0.008). This confirms that patient adaptation (i.e., familiarity with the procedure in the second session) was a significant confounder, which was appropriately controlled for by the LMM. Second, the analysis showed no significant carryover effect for any primary hemodynamic or satisfaction outcome (*p* > 0.05), confirming that our 1-week washout period was sufficient. Most importantly, even after controlling for these confounding period effects, the modality effect (3D virtual glasses vs. desktop) remained highly significant (*p* < 0.001). This confirms that the superior efficacy of 3D virtual glasses is a robust finding and not an artifact of patient adaptation.

The clinical implications of these findings relate directly to diagnostic accuracy and the avoidance of false-positive diagnoses. A primary goal of ICI and AVSS is to induce sufficient cavernosal smooth muscle relaxation, for which the EHS serves as the key clinical surrogate. While we did not measure smooth muscle tone directly, our study’s EHS results, as detailed in Table 1, provide strong, statistically significant evidence on this point. Patients were 6.68 times more likely to achieve a functional erection (83.6% of sessions) when using 3D virtual glasses compared to the desktop (43.3% of sessions) (odds ratio 6.68, 95% confidence interval [2.54, 17.55], *p* < 0.001). This markedly higher rate of successful functional erections is consistent with superior smooth muscle relaxation and strongly suggests a corresponding and significant reduction in false-positive diagnoses. Such diagnostic accuracy is of paramount importance. A misdiagnosis of vasculogenic ED can lead to patients being recommended for unnecessary and potentially hazardous invasive treatments. Conversely, the failure to accurately identify psychogenic ED may deprive patients of the opportunity to receive the most appropriate therapies, such as psychiatric counseling or psychotherapy. Ultimately, diagnostic variability undermines the reliability of the test for both clinicians and patients.

The analysis of patient anxiety provided a nuanced but critical finding regarding anticipatory anxiety. Due to a significant carryover effect identified in the LMM analysis for STAI-S scores (Table 1), a parallel-group analysis was performed (Table 2). This analysis revealed that while post-procedural anxiety levels after the first session were comparable between the desktop (Mean STAI-S = 25.47) and 3D virtual glasses (Mean STAI-S = 23.13) groups (*p* = 0.263), a highly significant difference emerged before the second session (*p* < 0.001). Patients who had first experienced the traditional desktop modality (Group 1) reported markedly higher anxiety (Mean STAI-S = 38.13) about their upcoming test compared to those who had first experienced 3D virtual glasses (Group 2) (Mean STAI-S = 25.87). This demonstrates that the immersive modality not only improves immediate satisfaction but also significantly lessens the psychological burden and dread of subsequent examinations, a critical factor for patient compliance in repeated follow-up.

The Samsung Gear VR utilized in our study represents a significant technological advancement over the head-mounted displays used in prior research. While previous studies demonstrated that even older wearable monitors could reduce anxiety by blocking external stimuli, these devices were often limited to simple 2D images or rudimentary 3D capabilities [16,22,23]. Conversely, our device provided a fully immersive, stereoscopic 3D experience. This enhanced level of immersion likely contributed to the superior outcomes observed in our trial through a dual mechanism: it not only minimized patient awareness of the clinical environment but also delivered a more potent and realistic form of sexual stimulation. This technological evolution is ongoing, and next-generation mixed-reality (MR) systems, such as the Meta Quest 3 (Meta Platforms, Inc.; Menlo Park, CA, USA), may offer even greater immersion, potentially leading to further improvements in clinical outcomes.

The primary strength of our study is its randomized crossover design, which minimized the influence of interpatient variability and increased the statistical power of the results. Another strength is the objective assessment of patient-reported outcomes, including satisfaction, willingness to undergo repeated procedures, and anxiety levels.

Despite these strengths, this study has some limitations. First, our study’s sample size is a limitation. Our a priori power analysis, based on a conservative estimate from the Carneiro et al. study, indicated a required minimum of 60 patients. Our enrollment precisely met this target. While this sample size was sufficient to detect significant differences in our primary hemodynamic outcomes, it remains relatively modest. This modest sample limits the generalizability of our findings and, importantly, lacks the statistical power needed for robust subgroup analyses. Second, and related to this limitation, our study did not exclude patients with comorbidities known to affect erectile function, such as diabetes mellitus or major psychiatric disorders, which could act as potential confounders. While our rigorous crossover design (where each patient acts as their own control) helps mitigate the impact of these baseline differences, a larger-scale trial would be necessary to validate our findings and conduct preplanned subgroup analyses to determine if the benefits of VR-AVSS vary by these patient characteristics. Third, the single-center design may affect the external validity of our findings. Furthermore, this study did not include subgroup analyses based on patient characteristics such as age, baseline IIEF scores, or anxiety levels. Such post hoc analyses were deemed inappropriate, as the current sample size would lack sufficient statistical power, thereby increasing the risk of yielding spurious Type I or Type II errors. Consequently, identifying which specific patient populations derive the greatest benefit remains a key objective for future, adequately powered investigations.

In addition to these research limitations, the broader clinical adoption of VR-AVSS also faces several practical implementation hurdles that must be addressed. First, the initial cost associated with acquiring VR headsets compared to conventional desktop monitors represents an important consideration. Second, as the device is shared among multiple patients, clear and standardized protocols for cleaning and infection control for the headset, which comes into direct contact with the face, are essential. Third, the issue of content curation. For consistency, this study provided the same video material to all participants; however, future research may be warranted to explore whether personalized content, tailored to individual patient preferences, could further enhance hemodynamic responses and satisfaction. Finally, headset tolerance in older patients is a key consideration. Given the average age of patients with ED, some may experience discomfort from the weight or unfamiliarity of the VR device. While headset tolerance was not reported as a significant issue in our study cohort, it remains an important factor for the widespread clinical adoption of this technology.

Therefore, while this study provides strong foundational evidence for the superiority of the VR modality, future large-scale, multicenter trials are warranted to validate these results and confirm their broader applicability. Building upon this validation, future investigations should delineate the modality’s efficacy in specific patient cohorts, particularly those with a significant psychogenic component to their ED, such as younger patients. To augment the robustness of these assessments, subsequent studies could incorporate objective physiological markers of sympathetic tone, such as heart rate variability, to corroborate subjective outcomes from the STAI. Finally, another pertinent research trajectory involves refining the interventional parameters; for instance, studies could investigate whether personalized visual stimuli yield superior hemodynamic and psychometric outcomes.

The findings of this study indicate AVSS with 3D virtual glasses, or next-generation VR/MR systems, are essential tools for ensuring accurate PDDU examinations. Clinically, these results support the integration of VR-AVSS into diagnostic guidelines as a standard option for patients at high risk of anxiety-induced false-positive results. The immersive modality could also be applied to other urological evaluations requiring maximal erection, such as RigiScan monitoring or the precise measurement of penile curvature in patients with Peyronie’s disease.

Nonetheless, a fundamental limitation persists in that the procedure requires the direct presence of a physician, inherently compromising patient privacy and potentially preventing complete relaxation. Therefore, the future of this field may lie in novel technologies. Wearable ultrasound patches, for example, could allow for private, home-based hemodynamic monitoring [27,28]. When combined with VR-AVSS, such a system could enhance diagnostic accuracy and expand opportunities for personalized treatment in patients with ED.

## 5. Conclusions

AVSS with 3D virtual glasses during PDDU is associated with superior erectile responses that decrease the incidence of false-positive results and improve patient satisfaction while significantly lowering anticipatory anxiety for subsequent procedures. This modality represents a significant improvement over traditional methods for PDDU-based evaluation of ED. Nonetheless, this study has some limitations, including a relatively small sample size and a single-center design, which may limit the generalizability of our findings. Further large-scale multicenter studies are needed to confirm these results, and continued research into novel technologies is warranted to enhance diagnostic accuracy.

## Figures and Tables

**Table 1:** Comparison of outcomes between modalities using mixed-effects models ^1^ and GEE ^2^.

	Modality	Adjusted Mean or Percentage (%)	Net Effect of 3D Virtual Glasses	95% Confidence Interval (CI) for Effect	*p*-Value (Modality Effect)	*p*-Value (Period Effect)	*p*-Value (Carryover Effect)
RI ^1^	Desktop	0.87	+0.03	[0.22, 0.44]	<0.001	0.011	0.796
	Virtual Glasses	0.90					
PSV ^1^ (cm/s)	Desktop	29.30	+5.97	[4.84, 7.10]	<0.001	0.008	0.800
	Virtual Glasses	35.27					
EDV ^1^ (cm/s)	Desktop	3.65	−0.29	[−0.53, −0.07]	0.013	0.078	0.927
	Virtual Glasses	3.36					
Satisfaction ^1^ (VAS)	Desktop	3.47	+2.13	[1.71, 2.55]	<0.001	0.748	0.194
	Virtual Glasses	5.60					
Willingness to undergo a repeat procedure ^1^ (VAS)	Desktop	3.30	+3.30	[0.93, 1.67]	<0.001	0.365	0.201
	Virtual Glasses	4.60					
STAI-S ^1^ (Anxiety)	Desktop	25.67	+4.96	[3.31, 6.62]	<0.001	<0.001	<0.001
	Virtual Glasses	30.63					
EHS ^1^ (Mean Score)	Desktop	2.40	+0.83	[0.64, 1.03]	<0.001	0.310	0.703
	Virtual Glasses	3.23					
Functioncal Erection ^2^ (EHS ≥ 3)	Desktop	43.3%	Odds Ratio 6.68	[2.54, 17.55]	<0.001	0.829	0.602
	Virtual Glasses	83.6%					

^1^ Analysis Method: Linear Mixed-Effects Model (LMM). ^2^ Analysis Method: Generalized Estimating Equations (GEE). EDV, End-diastolic velocity; EHS, Erectile hardness score; PSV, Peak systolic velocity; RI, Resistive index; VAS, Visual analog scale; STAI-S, State-Trait Anxiety Inventory-State.

**Table 2:** STAI-S score comparison by group.

	Group	STAI-S	*p*-Value
After Session 1	1	25.47 ± 6.82	0.263
	2	23.13 ± 3.93	
Before Session 2	1	38.13 ± 5.60	<0.001
	2	25.87 ± 3.07	

Continuous variables are presented as mean ± standard deviation. STAI, State-Trait Anxiety Inventory.

## Data Availability

The data presented in this study are available upon request from the corresponding author owing to legal issues regarding privacy.

## Abbreviations

The following abbreviations are used in this manuscript:

ED	Erectile dysfunction
PDDU	Penile duplex Doppler ultrasound
ICI	Intracavernous injection
AVSS	Audiovisual sexual stimulation
VR	Virtual reality
MR	Mixed reality
PDE5	Phosphodiesterase type 5
PSV	Peak systolic velocity
EDV	End-diastolic velocity
RI	Resistive index
EHS	Erectile hardness score
IIEF	International Index of Erectile Function
STAI	State-trait anxiety inventory
VAS	visual analog scale
